# Dichotic listening tests in students with learning disabilities

**DOI:** 10.1590/S1808-86942010000200018

**Published:** 2015-10-19

**Authors:** Fábio Henrique Pinheiro, Adriana Marques de Oliveira, Ana Cláudia Vieira Cardoso, Simone Aparecida Capellini

**Affiliations:** MSc student in Education - School of Sciences and Phylosophy - Paulista State University - FFC/UNESP-Marília - SP / Brasil, Speech and Hearing Therapist; MSc student in Education - School of Sciences and Phylosophy - Paulista State University - FFC/UNESP-Marília - SP / Brasil, Speech and Hearing Therapist; PhD in Human Communication Disorders - Federal University of São Paulo - UNIFESP -São Paulo - SP / Brasil. Professor - Department of Speech and Hearing Therapy - School of Sciences and Phylosophy - Paulista State University - FFC/UNESP-Marília - SP / Brasil - PhD; Assistant Professor; PhD in Medical Sciences - School of Medical Sciences of the State University of Campinas - FCM/UNICAMP- Campinas - SP / Brasil. Professor - Department of Speech and Hearing Therapy and of the Graduate Program in Education - School of Sciences and Phylosophy - Paulista State University - FFC/UNESP-Marília - SP / Brasil, Assistant Professor, PhD

**Keywords:** learning, evaluation, hearing disorders

## Abstract

Auditory processing disorder is a clinical entity that may be associated with several neuropathological disorders - learning disabilities among them.

**Aim:**

to characterize and compare the performance of students with and without learning disabilities in speech tests with and without background noise, dichotic listening tests, alternating dissyllable test.

**Materials and methods:**

40 students of both genders, ranging from 8 to 12 years of age participated in this study. They were divided in two groups: GI – 20 students with learning disabilities and GII – 20 students with good academic performance matched according to gender, age and education with GI. The evaluation consisted of basic audiological evaluation and applying dichotic listening tests, alternating disyllable test and speech test in noise.

**Study design:**

this is a cross-sectional study with a historical cohort.

**Results:**

the students of GI presented inferior performance compared to Group II (GII), both on dichotic listening tests and on alternating disyllable tests, and performance with no statistically significant difference on the speech in noise test.

**Conclusion:**

The evidence found suggests that the group of children with learning disabilities shows inferior performance compared to the group without problems, reflecting difficulties on the processing of auditory information.

## INTRODUCTION

In order to properly process auditory information, the sounds must be detected and interpreted, that is, the acoustic stimuli must be received by the peripheral auditory system, neutrally coded and turned into internal representations which will be analyzed and integrated by the Central Auditory System^1^.

This way, the acoustic stimulus must go through an auditory processing which involves mechanisms and processes pertaining to the auditory system which are responsible for: sound lateralization and localization, auditory discrimination, recognition of hearing temporal aspects and patterns - including resolution, masking, temporal integration, ordering and hearing performance with competitive and degraded acoustic signals^2^^,^^3^.

Auditory processing is associated to what we do with what we hear4. Therefore, it is not enough to have normal auditory thresholds; it is necessary that the acoustic signal be analyzed and interpreted in order to turn into a meaningful message. Thus, the auditory processing disorder is associated to difficulties in the processing of information at the central nervous system shown by a low performance in one or more auditory skills^2^.

The auditory processing disorder is a clinical entity of difficult diagnosis, because it can be associated with numerous human communication disorders - learning disorders among them^5^. Some authors^6^^,^^7^ consider the following as main causes of auditory processing disorders: neurological conditions, central nervous system development delays and the coexistence of other developmental disorders. The auditory processing disorder can also be caused, besides hereditary factors, by recurrent otitis media, usually during the time of auditory pathway development and peripheral hearing loss stemming from the sensorial privation it causes.

The auditory processing disorder creates communication difficulties with background noise, difficulties to understand jokes, attention reduction towards auditory messages, difficulties to understand reading and using expressive language (language rules). This disorder also causes difficulties in the production of certain speech sounds / r / and / l /, besides low school performance, although the intelligence level presented by the school-aged children is normal^8^.

The auditory processing disorder is associated with learning disorders and with the caveat that these are distinctive clinical entities, and we notice the possibility they have of coexisting^9^^,^^10^. Data indicates that the prevalence of learning disorders in school-aged children varies between 5 and 10% in the North-American population, and as far as the auditory processing disorder is concerned this value is of 2–3%^11^. Nonetheless, in Brazil we lack studies on the prevalence of auditory processing disorders in school aged-children, especially in relation to learning disorders.

Based on the aforementioned, this paper aimed at characterizing the auditory performance of school-aged children with learning disorders in the dichotic listening tasks presented and to compare the auditory performance of school-aged children with and without learning disorders in the tasks of speech with noise, digits dichotic and alternative dissyllabic dichotic (SSW) tests.

## MATERIALS AND METHODS

This study was carried out after having been approved by the Ethics in Research Committee under protocol # 2595/2007.

We used the following inclusion criteria:

– Signing the Free and Informed Consent form;
– School-aged children with normal sight, hearing and cognitive performance;
– School-aged children with learning disorders proved by means of a neuropsychological, neurological and audiological exam;

As exclusion criteria we used the following:

– Not having signed the Free and Informed Consent Form;
– School-aged children with multidisciplinary diagnosis of Development Dyslexia;
– School-aged children with sight, hearing and cognitive performance below normal standards;
– Other genetic or neurological syndromes;

We had 40 school-aged children from both genders, in the age range between 8 and 12 years participating in this study, and they were broken down into two groups:

Group 1 (G1): made up of 20 school-aged children with a multidisciplinary diagnosis of Learning Disorder.

Group 2 (G2): made up of 20 school-aged children with good school performance who passed the municipal public school, and were paired according to gender, age range and education as in G1.

Data collection started after the parents or guardians signed the Free and Informed Consent Form on behalf of the children.

The children were submitted to the following evaluation procedures:

• Audiological assessment:
– Basic audiological evaluation and the behavioral evaluation of the auditory processing were carried out in a sound-treated booth, in accordance with the standards. For threshold tonal audiometry and logoaudiometry we used the GSI 61 (ANSI S *3.6* −1989 and S3.43 −1992 Standards) audiometer with a TDH – 50 headphones. Hearing thresholds were studied by means of the descending technique for threshold attainment, in the sound frequencies of 1K, 2K, 3K, 4K, 6K, 8K, 500 and 250 Hz. The speech recognition threshold (SRT) was used to confirm the mean values for the sound frequencies of 500, 1K and 2K Hz.

As far as audiological normal values are concerned, we considered the following parameters: have auditory threshold for pure tone in the following sound frequencies: 250, 500, 1K, 2K, 3K, 4K, 6K and 8K Hz between 0 and 15 dBHL(ANSI 69 standard), according to a criterion proposed by Northern & Downs (1984).

We must stress that the school children in this study were referred to us after having been seen by an ENT physician.

• Digit dichotic listening, verbal dichotic listening - SSW and speech under noise tests)

The evaluation carried out through these tests was done with a two-channel GSI-61 audiometer, to which we coupled a Sony CD Player. We used a CD with the following tests: speech under noise, digits dichotic test and alternate dissyllable dichotic test (SSW).

The speech under noise test was carried out in a +5dB noise to signal ratio, and the noise was presented only contralateral, having one ear tested at a time.

The digits dichotic test aimed at assessing the individuals' skills associated with group acoustic signal components in background-figures and identifies them. The test uses a list of 20 digit pairs. Initially, four digits are dichotically presented, in other words, presented to both ears, and the individual must verbally repeat the digits presented. Following the procedure, the digits pair list is once again presented to him in two occasions and on the second presentation the individual must repeat the digits presented to the right ear and on a third time, to the left ear, and these are the listening times guided to the right and left ears.

The Portuguese-Language Alternate Dissyllable Dichotic test (SSW): was carried out at an intensity of 50dBSL and has 40 items. Each item is made up of 4 words consisting of two pairs of paroxytone disyllables. The child should repeat what he is hearing, following the word presentation order.

In order to do the statistical analysis, we used the SPSS (Statistical Package for Social Sciences), version 13.0, with a 5% significance level. We used the Mann-Whitney and the Wilcoxon Signaled Posts tests in order to check for possible differences between pre and post-testing times, considered during the assessment of each group.

We used 5% (0.050) as the significance level used in the statistical tests, in other words, when the calculated significance value (p) was below 5% (0.050), we noticed a “statistically significant” association, marked with an asterisk (*), and when the calculated significance value (p) was equal to or higher than 5% (0.050), we had a statistically non-significant association.

## RESULTS

Table 1 shows a graphical distribution of the mean, standard deviation and p-value in the Digits Dichotic Test.

**Table 1:** Graphical distribution of the mean, standard deviation and p-value in the Digits Dichotic Test.

Variable	GROUP	Mean	Standard deviation	p-value
RE	I	74,13	13,50	< 0,001*
	II	98,48	1,13	
LE	I	73,89	13,23	< 0,001*
		97,63	1,58	

Legend: RE: right ear; LE: left ear

We noticed that the mean performance of the group diagnosed with learning disorder (G1) was lower when compared to the mean levels observed in G2, both for the right and left ears, showing that G2 individuals had better performance in their skills regarding group acoustic signal components in figure and background noise and also to identify them.

After employing the Mann-Whitney statistical test, it was possible to observe a statistically significant difference in comparing the two groups, showing a superior performance by G2 individuals in both ears when compared to the mean value of correct answers provided by G1 individuals in characterizing their performance in the Digits Dichotic task.

**Graph 1:**
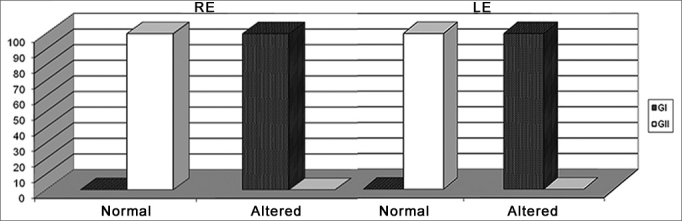
Classification of Alternate Digits Dichotic Test in Groups G1 and G2.

Graph 1 shows performance classification from G1 and G2 in the Digits Dichotic test.

RE: right ear, LE: left ear

We noticed that the school-aged children from G2, who did not have difficulties since they did not have alterations associated with the results from this test - suggesting that there are no alterations in relation to the background and figure auditory skills for verbal sounds and complex temporal ordering of verbal sounds.

Group 2, made up of school-aged individuals with learning disorders presented alterations associated with the auditory skills assessed in this test both for the right and left ears, having 100% of the children classified among those with alterations.

Table 2 shows the graphical distribution of the mean, standard deviation and p-value in the Alternate Dissyllable Dichotic test (SSW).

**Table 2:** Graphical distribution of the mean, standard deviation and p-value in the Alternate Dissyllable Dichotic Test (SSW)

Variable	GROUP	Mean	Standard deviation	p-value
RE	I	66,75	23,14	< 0,001
	II	96,25	3,39	
LE	I	69,00	15,00	< 0,001
		95,56	3,74	

Legend: RE: right ear; LE: left ear

We can notice that Group 1 presented performance mean values which were higher when compared to those from Group 1, for both ears, showing that G1 individuals had a higher performance in relation to this skill, and that during the test it is assessed verbally, with speech stimuli. G1 had higher correct-answer mean values when compared to individuals from G2 as they grouped background and figure acoustic component grouping and identifying them, later on referring to the sequence of words presented, following the presentation's order and shape. The statistical analysis carried out by means of the Mann-Whitney test showed statistically significant difference when comparing the two groups and the higher performance by G2 individuals in both ears, reflecting the difficulties in background figure, auditory attention and organization presented by Group 1 individuals.

**Graph 2:**
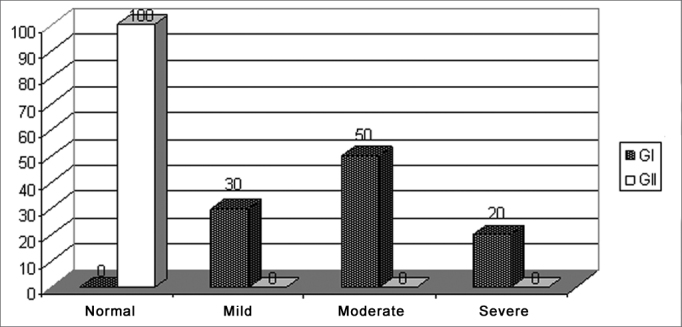
Classification of results from the Alternate Dissyllable Dichotic Test (SSW) in Portuguese language for G1 and G2.

GI: Group 1; GII: Group 2

Graph 2 shows the performance classification for G1 and G2 in the Alternate Dichotic Disyllables test (SSW).

We noticed that those children diagnosed with learning disorders who were part of the G1 had alterations in their hearing skills, and were classified as to their performance in this auditory processing test in the following way: 30% of them had mild alterations; 50% moderate alterations and 10% severe alterations. G2, made up of school-aged children without learning difficulties did not have alterations in regards of their figure and background auditory skills for verbal sounds and complex temporal ordering of verbal sounds, and 100% of them were classified as normal.

Table 3 shows a comparison between G1 and G2 regarding speech under noise tests.

**Table 3:** Comparing the classification of the speech under noise tests in Groups 1 and 2

GROUP	Classification		Total
	Normal	Altered	
I	19	1	20
	90,00	10,00	100,00
II	20	0	20
	100,00	0,00	100,00
Total	39	1	40
	97,5	2,5	100,00

We've noticed that 20% of the G1 children had alterations in regards of this skill, while G2 children did not show any alteration, and this was caused by the lack of alterations in the auditory processing of these individuals.

**Graph 3:**
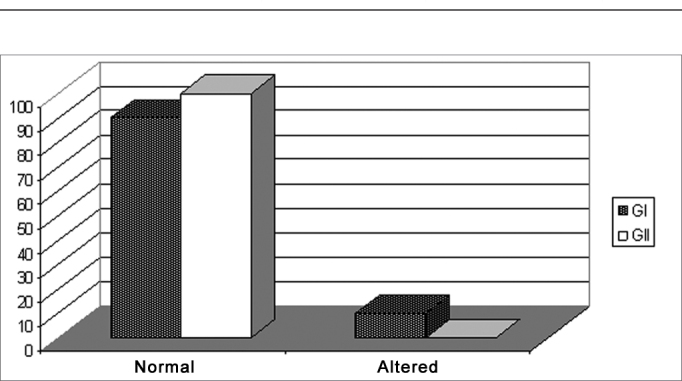
Classification of the Results from the Speech Intelligibility Test with ipsilateral competitive message (speech under noise) in groups G1 and G2 in percentages.

GI: Group 1; GII: Group 2

We noticed that in Graph 3, the G1 children diagnosed with learning disorders had alterations in their auditory skills, being classified in the following way: 90% of the individuals were within normal standards according to pre-established criteria^12^, and 10% we classified with some alteration also according to the aforementioned criteria. Group 2, made up of school-aged children without learning difficulties, did not show alterations in regards of this test, where 100% of the individuals were classified as normal.

## DISCUSSION

The results from the tonal threshold audiometry did not show alterations, thus corroborating with the literature which does not point to any direct relation between the auditory processing disorder and hearing loss, since for its characterization it is necessary to have normal auditory accuity^13^^,^^14^.

The results of the auditory processing tests made up of the Digits Dichotic and Alternate Dissyllable Dichotic tests showed a lower mean value of correct answers in both ears in the group of children diagnosed with learning disability. These findings corroborate the international literature which points out such difference ^15^^,^^16^ and can interfere directly on the skills which require handling speech information in the memory and which are necessary for reading and writing learning at school age, and this is seen among children with learning disorders^17^.

School-aged children with learning disorders have reduced response capacity facing the stimuli presented because of alterations in the development of auditory attention skills. These children have significant loss in this skill, and this is a fact which corroborates findings in the International Literature^18^.

The data obtained from the application of the Digits Dichotic Test (directional hearing stage), used in order to assess the figure-background skills for verbal sounds in sustained attention processes and selective attention, showed that the school-aged children with learning disorders had lower mean values of correct answers then those without these shortcomings. According to Table 1, the mean value of correct answers with the use of the right ear from the group with disorder was substantially lower, and the same happened when comparing the left ears from both groups. These data reinforce the idea that the school-aged children with learning disorders have alterations in their auditory attention skills and maintenance, which impair the figure-background skill for verbal sounds from these children.

Those children with learning disorders have prolonged concentration difficulties18 and, as a consequence, a loss in auditory information processing and perception, being it passed on by the professional in charge during the auditory testing or by the teacher in the classroom, and the result of this disability seen in the results of the tests applied.

Some studies have been carried out with the deployment of processing tests, such as the Digits Dichotic^19^ and the SSW dichotic task^20^ and have stressed the importance of its application in order to obtain data on the development of the auditory process in children and its contribution for the early detection of any disorder which could reflect on the social and academic lives of these individuals.

Moreover, the results from the auditory processing test are classified according to the degree of involvement and this type of classification is important, having seen that it allows for a treatment guidance towards the auditory difficulty detected, and allows for a better stimulation work to be performed in accordance to the individual's complaint^21^^,^^22^.

The data presented on Graph 1 shows 30% of the school-aged children with learning disorders with a mild processing impairment, 50% with moderate impairment and 20% severe. This data reflects the difficulty these children have when dealing with auditory information, and this difficulty can be more or less significant according to the degree of alteration found^12^.

The classification of the speech intelligibility test results speaks for itself and can not indicate disorder or alteration in the auditory processing since only 10% of the children studied have some kind of alteration, not reflecting the broader analysis of the data provided by the remaining tests.

The children with learning disorders who had figure-background and selective attention difficulties had problems in organizing the auditory information, as we can see in the tests applied, which was reflected in their results from the applied auditory tests.

We stress that the results from this study must be interpreted in a biased way, since the sample does allow for generalizations, requiring additional studied for better understanding the auditory processing assessment in school-aged children with learning disorders.

## CONCLUSION

Our findings allow us to infer that the performances of school-aged children with learning disorders in the digit dichotic and verbal dichotic listening tests was lower than the average from the group of children without learning disorders. Nonetheless, in the speech-under-noise test we did not find statistically significant differences in the performance of the two compared groups. These findings point to the fact that the group of school-aged children with learning disorders have alterations in their attention auditory skills, acoustic information integration, sequencing and organization of the acoustic signals and the figure-background acoustic signal for verbal sounds, which end up compromising its performance in the auditory processing tests.
